# Comparing the effects of serum GPER-1 and oxidant/antioxidant levels on retinopathy in patients with diabetes and healthy individuals: a pilot study

**DOI:** 10.5935/0004-2749.2021-0311

**Published:** 2022-07-04

**Authors:** Abdullah Beyoğlu, Ergül Belge Kurutaş, Yalçın Karaküçük, Ayşegül Çömez, Ali Meşen

**Affiliations:** 1 Department of Ophthalmology, Faculty of Medicine, Sutcu Imam Universty, Kahramanmaras, Turkey; 2 Department of Biochemistry, Faculty of Medicine, Sutcu Imam Universty, Kahramanmaras, Turkey; 3 Department of Ophthalmology, Faculty of Medicine, Selcuk Universty, Konya, Turkey

**Keywords:** Diabetic retinopathy, GPER-1, Estradiol, Progesterone, Oxidative stress, Oxidants, Retinopatia diabética, GPER-1, Estradiol, Progeste­rona, Estresse oxidativo, Oxidantes

## Abstract

**Purpose:**

This study aimed to determine the effect of serum G receptor-mediated protein-1 levels on the development of retinopathy in patients with diabetes in comparison with healthy individuals.

**Methods:**

The study enrolled patients with diabetic retinopathy (Group 1), patients without diabetic retinopathy (Group 2), and healthy individuals (Group 3). Levels of serum progesterone, serum G receptor-mediated protein-1, estradiol, oxidant/antioxidants, and thyroid-releasing hormones were analyzed and compared among the groups. Post-hoc analysis was performed to compare the subgroups in which significant differences were found.

**Results:**

Groups 1, 2, and 3 each included 40 patients. A significant difference was found among all groups in terms of serum G receptor-mediated protein-1, oxidant/antioxidant, and estradiol levels (p<0.01), but no significant difference was found in terms of thyroid-releasing hormone or progesterone (p=0.496, p=0.220, respectively). In the post-hoc analysis of the groups with significant differences, another significant difference was found among all groups for serum G receptor-mediated protein-1 and oxidant/antioxidant levels (p<0.05). Serum G receptor-mediated protein-1 and oxidant levels were positively correlated, whereas serum G receptor-mediated protein-1 and antioxidant levels were negatively correlated (r=0.622/p<0.01, r=0.453/p<0.01, r=0.460/p<0.01, respectively). The multiple regression analysis showed that increased levels of serum G receptor-mediated protein-1 may help prevent diabetic retinopathy.

**Conclusions:**

Serum G receptor-mediated protein-1 levels, which were the highest in the diabetic retinopathy Group, increased as the oxidant/antioxidant balance changed in favor of oxidative stress. This appears to be a defense mechanism for preventing neuronal damage.

## Abstract (pt)

**Objetivo:**

Esta pesquisa buscou determinar o im­pacto dos níveis de proteína G sérica no desenvolvimento da retinopatia em pacientes diabéticos, comparando-os a indivíduos saudáveis.

**Métodos:**

Foram incluídos, no estudo, 40 pacientes com retinopatia diabética (Grupo 1), 40 pacientes sem retinopatia diabética (Grupo 2) e 40 indivíduos saudáveis (Grupo 3). Os níveis hormonais de progesterona sérica, de proteína G sérica, estradiol, oxidante/antioxidante e hormônio liberado pela tireoide foram analisados e comparados. A análise post hoc foi realizada para comparar os subgrupos nos quais diferenças estatisticamente significativas foram encontradas.

**Resultados:**

Uma diferença significativa foi encontrada entre todos os grupos em termos de proteína G sérica, oxidante/antioxidante e níveis de estradiol (p<0.01), mas nenhuma diferença significativa foi encontrada em termos de hormônio liberado pela tireoide ou progesterona (p=0,496, p=0,220, respectivamente). Na análise post hoc dos grupos com diferenças estatisticamente significativas, outra diferença significativa foi encontrada entre todos os grupos para proteína G sérica e níveis oxidantes/antioxidantes (p<0,05). Os níveis de proteína G sérica e os níveis de oxidante foram positivamente correlacionados, enquanto os níveis de proteína G sérica e os níveis de antioxidantes foram negativamente correlacio­nados (r=0,622/p<0,01, r=0,453/p<0,01, r=0,460/p<0,01, respectivamente). A análise de regressão múltipla mos­trou que o aumento da proteína G sérica pode ajudar a prevenir a retinopatia diabética.

**Conclusões:**

Os níveis de proteína G sérica que eram mais altos no grupo de retinopatia diabética, aumentaram à medida que o equilíbrio oxidante/antioxidante mudou em favor do estresse oxidativo. Este parece ser um mecanismo de defesa para prevenir danos neuronais.

## INTRODUCTION

Diabetic retinopathy (DR) is among the most common complications of diabetes; it can present with symptoms such as exudate, hemorrhage, macular edema, microangiopathy, and neovascularization^(1)^. Worldwide, it is also the most common preventable cause of blindness^(2)^. DR is diagnosed by clinical signs of vascular pathologies in the retina, and it is divided into two main clinical stages, namely, proliferative DR (PDR) and nonproliferative DR (NPDR)^(1,3)^. Although pathologies such as microaneurysms, hemorrhages, and exudate are common during the NPDR stage, patients may be asymptomatic. However, in the PDR stage, serious outcomes such as intravitreal he­morrhages or tractional retinal detachment due to neovascularization may occur^(1,3,4)^. DR etiology involves many complex mechanisms. These include retinal ischemia (formed due to vascular pericytes and endothelial damage), glial cell dysfunction (caused by increased inflammatory mediators), and homeostasis disruption (due to higher levels of free reactive oxygen species [ROS] that increase as the oxidant/antioxidant balance changes)^(5-7)^.

G protein-coupled estrogen receptor-1 (GPER-1) is a transmembrane estrogen receptor localized in the endoplasmic reticulum (ER), which binds with high affinity to 17β-estradiol^(8,9)^. It is present in various body systems, including the reproductive, nervous, endocrine, immune, and cardiovascular systems^(10)^. GPER-1 expression was also found in the retina^(11)^.

Oxidative stress deteriorates the homeostatic balance, causing protein damage and, thus, ER stress. This damages cells and induces apoptosis^(12)^. However, previous studies have shown that GPER-1 activation causes ROS to decrease, which rapidly reduces the stress on the ER^(10,12)^.

Currently, various treatment methods (e.g., antivascular endothelial growth factor, steroid, and laser therapies) are used to reduce the oxidation and inflammatory mediators of DR pathogenesis^(6,13)^. However, retinal neurodegeneration could be an independent pathophysiological component of DR, and new molecular mechanisms should be investigated to detect early damage and initiate treatment^(1,14)^. Upon review of the extant literature, the present researchers did not find studies examining oxidative stress and GPER-1 levels in patients with diabetes in comparison with healthy participants.

Therefore, this study compared the serum GPER-1 levels and oxidative stress markers (malondialdehyde [MDA], catalase [CAT], and superoxide dismutase [SOD]) in patients with diabetes and healthy individuals.

## METHODS

### Study design and participants

This prospective study included patients with type 2 diabetes (with DR, [Group 1] and without DR [NDR, Group 2]) and a control group of healthy individuals (Group 3).

All participants provided written informed consent. The study was performed in compliance with the ethical principles of the Declaration of Helsinki, and approval for the research was obtained from the local ethics committee (Protocol no. 03-2018/20, 7, 2018).

The inclusion criteria were as follows: individuals with confirmed DR, had no NDR, and were confirmed to be healthy, without any systemic disease. Candidate participants were excluded from the study if they had glaucoma, ocular trauma sequelae, pathological myopia, NDR, a history of previous ocular surgery, endocrine disorders (e.g., thyroidopathy, adrenal gland disorders, and pituitary pathologies), and opacities interfering with fundus examination (e.g., corneal opacity, lens opacity, and vitreous cloudiness other than diabetic hemorrhage). Those who had received hormone replacement therapy or were addicted to alcohol and/or controlled substances were also excluded, as were premenopausal women (to avoid gender-related complexities). Fasting venous blood samples were taken from the participants, following a complete ophthalmological examination. In the serum obtained, levels of GPER-1, MDA, CAT, SOD, estradiol, thyroid-stimulating hormone (TSH), and progesterone were examined, and the groups were compared.

### Preparation of blood samples

Fasting blood samples (5 ml) were taken from the participants’ median cubital veins. Circadian variations were avoided by always drawing samples between 8:00 am and 9:00 am. Blood samples were allowed to clot before centrifugation.

### Serum

The blood samples were immediately centrifuged at 2,000 rpm for 10 min at 4°C. Supernatant serum was separately stored at 80°C before enzyme-linked immunosorbent assay (ELISA) was used to measure serum estrogen, progesterone, and GPER-1 levels.

### Biochemical analysis

Serum TSH, progesterone, GPER-1, and estradiol levels were measured using a quantitative sandwich ELISA method via a commercial kit (SEG 045 Hu, Cloud-Clone Corp., Houston, TX, USA) according to the manufacturer’s instructions.

SOD activity was measured in the samples according to the method described by Fridovich^(15)^. This method employs xanthine and xanthine oxidase to generate superoxide radicals, which react with p-iodonitrotetrazolium violet to form a red formazan dye, which is measured at 505 nm. The assay medium contained 0.01 M phosphate buffer, a 3-cyclohexilamino-1-propanesulfonic acid (CAPS) buffer solution (50 mM CAPS, 0.94 mM etheylenediaminetetraacetic acid [EDTA], saturated sodium hydroxide [NaOH]) with a pH of 10.2, a substrate solution (0.05 mM xanthine, 0.025 mM INT), and 80UL xanthine oxidase. SOD activity was expressed as U/mg protein.

CAT activity was determined by measuring the decrease in hydrogen peroxide concentration at 230 nm via Beutler’s method^(16)^. The assay medium contained 1 M Tris hydrochloride, 5 mM disodium (Na_2_) EDTA buffer solution (pH 8.0), 10 mM hydrogen peroxide, and a blood sample, creating a final volume of 1.0 ml.

MDA levels in the samples were measured with the thiobarbituric acid (TBA) test^(17)^. Each reaction mixture contained 0.1 ml of a blood sample, 0.2 ml of 8.1% sodium dodecyl sulfate, 1.5 ml of 20% acetic acid, and 1.5 ml of 0.8% TBA aqueous solution. The pH of the mixture was adjusted to 3.5, and the volume was increa­sed to 4.0 ml with distilled water. Then, 5.0 ml of an n-butanol and pyridine (15:1, v/v) mixture was added. The final reaction mixture was shaken vigorously. After centrifugation at 4,000 rpm for 10 min, the absorbance of the organic layer was measured at 532 nm.

### Statistical analysis

The data obtained were statistically analyzed using the IBM SPSS Statistics for Windows, version 22.0 (IBM Armonk, NY, USA). The data’s conformity to normal distribution was assessed via the Shapiro-Wilk test. Categorical data were analyzed using the chi-squared test. An analysis of variance was performed to compare the groups. The data in these group comparisons were normally distributed. Post-hoc analysis was performed to determine the difference between the significant variables. Continuous data were expressed as mean ± standard deviation (SD), and categorical data were presented as numbers (n) and percentages (%). The results were considered significant at p<0.05.

## RESULTS

Groups 1, 2, and 3 each included 80 patients. Table 1 presents the demographic data for the groups and information concerning the duration of diabetes. No significant differences were found between the groups in terms of age or sex (p=0.527, p=0.699, respectively). However, a significant difference was found for diabetes duration (p<0.01), and the DR group had the longest diabetes duration.

**Table 1:** Demographic and clinical characteristics of the groups

**Characteristic**	**Group 1** (n_1_**=40)** Mean + SD	**Group 2** (n_2_**=40)** Mean + SD	**Group 3** (n_3_**=40)** Mean + SD	p-value^*^
Age (years)	57.47 ± 6.40	56.02 ± 6.79	55.75 ± 8.52	0.527^*^
Gender (Male/Female)	19/21 47.5%/52.5%	21/19 52.5%/47.5%	20/20 50.0%/50.0%	0.699^*^
DM (years)	11.82 ± 5.95	6.1 ± 2.47	**N**	<0.01^*^

n_1_= Number of patients with diabetic retinopathy.

n_2_= Number of patients without diabetic retinopathy.

n_3_= Number of control participants.

DM= diabetes mellitus; SD, standard deviation.

* = *Chi-square test.*

P-value of <0.05 was considered significant (bold).

A significant difference was also found among all groups in terms of serum GPER-1, MDA, CAT, SOD, and estradiol (p<0.01), but no significant difference was observed among the groups in terms of progesterone and TSH (p=0.496, p=0.220, respectively). In the post-hoc analysis of parameters showing significant differences, a significant difference was found between GPER-1, MDA, CAT, and SOD in all groups, and a significant difference for estradiol was determined between Group 1 and Group 3 (p<0.01) (Table 2).

**Table 2:** Comparison of groups in terms of GPER-1, hormones, and oxidative markers

	**Group 1**^a^ **n**_1_**=40** Mean ± **SD**	**Group 2**^b^ **n**_2_**=40** Mean ± **SD**	**Group 3**^c^ **n**_3_**=40** Mean ± **SD**	**p-value**
GPER-1(ng/ml)	0.501 ± 0.09^b,c^	0.455 ± 0.06^a,c^	0.379 ± 0.03^a,b^	**<0.01**
Estradiol (ng/ml)	56.27 ± 5.85^c^	58.27 ± 5.14	62.65 ± 11.84^a^	**0.02**
Progesterone (ng/ml)	11.56 ± 1.12	11.66 ± 0.77	11.92 ± 0.83	0.220
TSH (ng/ml)	1.29 ± 0.10	1.28 ± 0.06	1.27 ± 0.09	0.496
MDA (nmol/mg prt)	4.97 ± 1.38^b,c^	2.97 ± 0.56^a,c^	1.92 ± 0.53^a,b^	**<0.01**
CAT (**µ**/mg prt)	93.09 ± 5.98^b,c^	122.25 ± 9.91^a,c^	174.82 ± 11.33^a,b^	**<0.01**
SOD (**µ**/mg prt)	0.79 ± 0.11^b,c^	1.16 ± 0.19^a,c^	1.55 ± 0.28^a,b^	**<0.01**

n_1_/n_2_/n_3_= Number of groups subject.

CAT= catalase; GPER-1= G protein-coupled estrogen receptor-1; MDA, malondialdehyde; SOD= superoxide dismutase; SD= standard deviation; TSH= thyroid-stimulating hormone.

Groups were coded with letters a, b, and c. One-way analysis of variance test was used to compare the means. Tamhane’s T2 post-hoc analysis was performed in paired comparison. Significance was p<0.05 (bold). In paired comparisons, the superscript letters indicate the group in which there was a significant difference between them.

**Figure 1:**
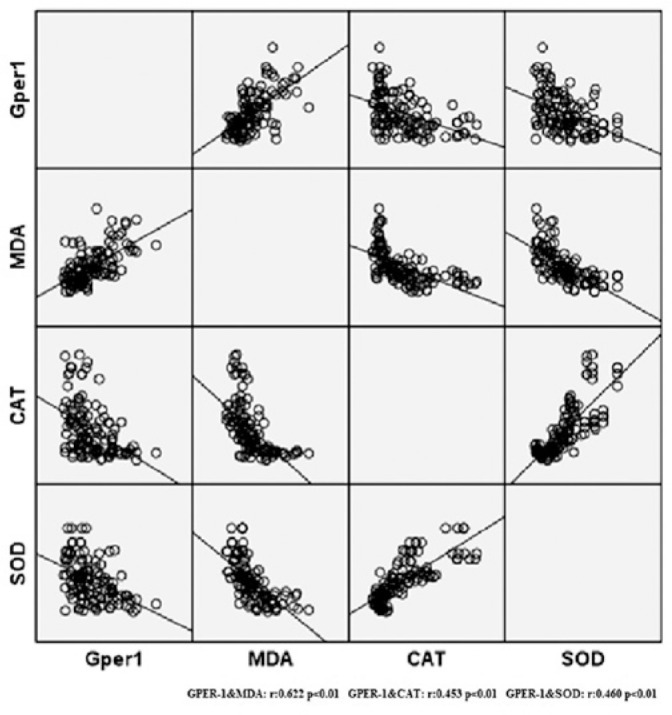
Correlation of GPER-1 with MDA, CAT, and SOD. GPER-1, G protein-coupled estrogen receptor-1; MDA, malondialdehyde; CAT, catalase; SOD, superoxide dismutase.

In the correlation analysis between GPER-1 and oxidant/antioxidant levels, GPER-1 correlated positively with MDA and negatively with SOD and CAT (r=0.622/p<0.01, r=0.453/p<0.01, r=0.460/p<0.01, respectively) (Figure 1).

Multivariate regression analysis was performed to assess the researchers’ model, which considers GPER-1 (a possible retinopathy preventative) alongside age, sex and diabetes duration (factors known to influence DR development). This analysis determined that GPER-1 and diabetes duration were statistically significant factors in DR pathogenesis (Table 3).

**Table 3:** Multivariate logistic regression analysis in the model created with GPER-1, age, gender, and duration of diabetes

	**p-value**	**OR**	**95% Cl**
Age (years)	0.418	0.962	0.875-1.057
Gender			
(Male/Female)	0.423	0.633	0.207-1.937
DM (years)	<0.01^*^	1.531	1.284-1.825
GPER-1 (ng/ml)	0.045^*^	1.023	1.012-1.943

CI= confidence interval; DM= diabetes mellitus; GPER-1= G protein-coupled estrogen receptor-1; OR= odds ratio.

P-value of <0.05 was considered significant.

* Bold.

## DISCUSSION

The existing treatment strategies for DR, including intravitreal pharmacological agents, laser photocoagulation and vitreous surgery, aim to manage microvascular complications. However, inadequate response to these treatments indicates the presence of other underlying mechanisms^(5-7)^. A growing body of laboratory and clinical evidence suggests that inflammation and retinal neurodegeneration may play a role as independent pathogenesis pathways in DR^(1,6)^.

Retinal neurodegeneration can occur even without DR development^(1)^. In diabetic animals, an increase in proapoptotic molecules triggers apoptosis in neurons, which leads to retinal thinning before DR development^(6)^. It has also been suggested that neuronal degeneration significantly increases with the formation of mitochondrial damage and oxidative stress due to high glucose exposure^(12)^. Thus, neuronal damage may be reduced by suppressing oxidative stress^(1,12)^.

GPER-1 is a rapidly acting, membrane-bound estrogen receptor, independent of gene regulation^(8,9)^. Although GPER-1 has been reported to mediate more than one type of estrogen activity in vivo, growing evidence indicates that GPER-1 also has gender-independent effects^(18)^. Evidence shows that GPER-1 plays a crucial role in metabolic regulation (lipids and glucose homeostasis, insulin production and action, etc.), blood pressure, and immune functions^(9,10,18)^. Previous studies have also indicated that GPER-1 activation increases the efficiency of glucose transporters, reduces oxidative stress products, and regulates the inflammatory response; thus, it plays a neuroprotective role^(1,11,12)^.

In patients with diabetes, the oxidant/antioxidant balance changes in favor of oxidation due to increased oxidative stress in the body^(19)^. Tawfik et al.^(20)^ revealed that increased oxidative stress impairs the blood-retinal barrier and induces apoptosis in the retinal neurons and glial cells. As a vicious circle, ROS levels increase as diabetes duration lengthens and diabetes complications occur. In the current study, a significant difference was found among Groups 1, 2, and 3 regarding GPER-1 and ROS. The incidence of diabetes and risk of developing complications increase as ROS levels rise. Moreover, serum GPER-1 levels rise. Therefore, the present researchers assert that GPER-1 levels rise in response to higher ROS levels.

Mancino et al.^(21)^ showed that mitochondrial ROS production is increased in hyperglycemia and suggested that mitochondrial abnormalities have irreversible consequences. They demonstrated that such mitochondrial ROS production continues even if a patient shifts to normoglycemia. This phenomenon has been termed the “metabolic memory” or “inheritance effect.” Through in vitro experiments, Wu et al.^(22)^ showed that retinal pericytes, which are exposed to hyperglycemia, continue to overproduce ROS, even in a normoglycemic environment. This situation supports the abovementioned results. In the present study, the highest ROS levels were found in individuals with DR, revealing their increased risk of complications. GPER-1 levels also increased to protect against ROS formation and consequences. This finding is supported by the positive correlation between SOD and CAT and GPER-1 and the negative correlation between MDA and GPER-1.

The mean estradiol level in the diabetic group differed significantly from that in the control group. The present authors believe that the GPER-1 levels were higher in patients with diabetes than in healthy controls (being highest in individuals with DR), indicating that they are produced to prevent cellular damage and apoptosis. They authors also think that GPER-1 is upregulated as estrogen levels decrease in patients with diabetes.

Retinal neurodegeneration begins before clinical retinopathy develops; thus, it is crucial to take earlier measures to prevent this progression. In this study, serum GPER-1 levels began to rise before DR development and reached their highest levels in patients with DR, indicating that GPER-1 is produced in response to ROS increase. This could be an important finding for preventing neurodegeneration. Han et al.^(23)^ demonstrated that G-1, which is a GPER-1 agonist, increases the viability of microglia in neuronal damage. This beneficial effect on microglia is reduced with G-15, which is a GPER-1 antagonist. Li et al.^(10)^ suggested that activating GPER-1 reduces ROS production by decreasing mitochondrial damage and, thus, increasing neuronal survival.

This study has several limitations. First, to minimize hormonal differences between the studied male and female participants, the researchers did not include premenopausal women in the sample. Second, they also did not measure the amounts of serum oxidative stress molecules in the blood samples by interfering with GPER-1 agonist agents, such as G-1. Third, subgroup analysis of diabetic retinopathy was not performed. Nevertheless, this is the first study to reveal the correlation between serum GPER-1 levels and oxidative stress molecules in patients with DR. Thus, it will light the way for further studies and novel treatments.

In conclusion, GPER-1 appears to be a remarkable receptor in following up the disease progression of patients with diabetes, detecting DR at an early stage, and preventing neuronal damage. Unlike natural estrogen or conjugated estrogens, applying GPER-1-specific medications in G-1-like retinal cells devoid of other endocrinological effects could be a potential therapy for early DR intervention and for unresponsive DR in combination with existing treatments. To explore this possibility, further studies, with larger samples, are needed.
